# Supplementary material for: Are carbon emissions trading and green financial instruments synergistic? -Comprehensive quantitative research based on content analysis
Source: PLoS One. 2024 Mar 7;19(3):e0298601. doi: 10.1371/journal.pone.0298601 (PMC10919601; doi:10.1371/journal.pone.0298601)
Supplement: S1 Data — (ZIP) [file pone.0298601.s001.zip › Data/地级市Z_F工作报告.zip!/║╙▒▒/│╨╡┬╩╨/└┤╫╘╣1⁄2╓┌║┼í╛┬φ┐╦╩2╛▌═°í┐_2018.docx]

各位代表：

现在，我代表市人民政府，向大会报告工作，请予审议，并请各位市政协委员和列席人员提出意见。

一、2017年工作回顾

刚刚过去的2017 年，全市上下认真学习宣传贯彻党的十九大精神，全面落实中央、省委省政府和市委各项决策部署，紧紧围绕“12345”总体思路目标，攻坚克难，砥砺前行，较好完成了市十四届人大一次会议确定的目标任务，为建设新时代生态强市、魅力承德奠定了坚实基础。

经济发展取得新成绩。坚持稳中求进工作总基调，经济整体回升向好。全市实现生产总值1618.6 亿元，增长7.1%；固定资产投资完成1732.5 亿元，增长7.2%；规上工业实现增加值563.9 亿元，增长5%；全部财政收入完成177.1亿元，增长20.5%；公共财政预算收入完成89.2亿元，增长8.6%。以上指标增速全省排位均居上游。大力实施重点项目建设“33591”工程，承钢钒钛高强冷轧板、青松岭文化旅游度假区等一批事关全局、支撑作用明显的重大项目顺利推进。农业稳步发展，粮食总产量达到138.4万吨，农业产业化经营率达到69.7%，功能农业培育取得新进展。经济发展内生动力进一步增强，现代服务业加快发展，消费对经济增长贡献率达到56.6%，第三产业税收贡献超出第二产业5.5 个百分点。

转型升级迈出新步伐。坚持绿色发展不动摇，积极培育新动能。十大绿色产业加快发展，绿色产业增加值占GDP 比重达到35%。高新技术产业增加值增长17.9%，高出工业12.9 个百分点。坚定不移去产能，圆满完成压减炼钢产能190 万吨、炼铁产能104 万吨、煤炭产能48 万吨目标任务。加快传统产业改造提升，工业技改投资增长30%，新增规上工业企业55 家。大力培育大智移云产业生态，德鸣大数据小镇、华明服务外包产业园等项目加快建设，博彦科技、软通动力等软件外包企业落户承德高新区，承德县高新区列入国家新型工业化（大数据）产业示范基地。成功举办首届全市旅游产业发展大会，一批旅游业新景区、新业态应运而生，全市游客接待量5796 万人次、旅游总收入679 亿元，分别增长25%和35%，荣获全国“厕所革命”十大优秀城市奖。

脱贫攻坚取得新进展。坚持精准扶贫精准脱贫，创新实施“八措并举”组合拳，全力推动脱贫攻坚向纵深发展。16.25 万贫困人口实现稳定脱贫，“政银企户保”“三零”扶贫新模式在全国全省推广。大力推进产业扶贫，食用菌、林果、马铃薯等九大特色产业，帮助12.1 万困难群众摆脱贫困、走向富裕。全国产业扶贫现场会在平泉召开，围场列入国家农业可持续发展试验示范区。易地扶贫搬迁“两区同建”完成45 个安置小区、1.01 万人的新居建设任务。积极实施万户阳光扶贫工程，建成村级电站131 个、户用光伏1.24 万个，带动2 万“三无”特困户实现稳定增收。全面推开“632”医疗保障救助扶贫模式，为68.66 万人次困难群众增报医疗费用2.6 亿元。扎实推进美丽乡村建设，建设省级美丽乡村精品片区4 个，围场、滦平、隆化等县的美丽乡村实现成线连片，农村人居环境显著改观。

生态文明建设达到新水平。习近平总书记对塞罕坝建设者感人事迹作出重要指示，塞罕坝被联合国授予“地球卫士奖”，被省委省政府授予“生态文明建设范例”，全国掀起学习弘扬塞罕坝精神热潮。坚持全民共治、源头防治，打赢蓝天保卫战，PM2.5 平均浓度35 微克、下降12.5%，提前三年达到国家二级标准。市区优良天数达到291 天，继续保持京津冀前列。持续开展国土绿化行动，新增造林绿化62.5 万亩，森林覆盖率达到57.67%，成功创建国家森林城市。加大矿山整治修复力度，完成复垦披绿、取缔关闭、绿色改造矿山各100 家。加强水污染防治，市县乡村四级全部建立“河长制”。全面取缔潘家口水库网箱养鱼，潘家口水库水质从Ⅴ类提升至Ⅲ类。系统推进生态建设体制机制改革，《水源涵养功能区保护条例》立法工作顺利推进。启动国家绿色发展战略环评和“三线一单”试点，对丰宁、兴隆开展领导干部自然资源资产离任审计，在围场县实施GEP 核算体系试点，承德特色的生态文明建设法制和制度体系加快构建。

协同发展实现新突破。认真落实京津冀协同发展重大国家战略，在对接和服务京津中加快发展。实施与京津合作项目295 个，完成投资610亿元。天津中德应用技术大学承德分校项目加快建设。交通基础设施一体化全面提速，京沈高铁承德段全线铺轨，承平高速即将全面开工。承德机场正式通航，开通6 条航线、通达7 个城市，旅客吞吐量达到14.6 万人次，实现了承德人民多年翘首以盼的“蓝天梦”。深入实施“一带三区”城市发展战略，加快推进“五城联创”，成功争创首批省级文明城市，并再获全国文明城市提名资格，围场、滦平和平泉三县（市）荣获首批省级文明县城和文明城市称号，平泉成功获批县改市。全市常住人口城镇化率达到50.7%，中心城区辐射力增强，县城建设提质提速，协同发展承载力进一步提升。面向珠三角开展精准招商，中美友谊示范农场建设正式启动，对外开放深度广度进一步拓展。

改革创新释放新活力。坚持改革引领、创新驱动，发展活力明显增强。继续深化“放管服”改革，取消下放行政许可事项11 项，市县行政审批局全部挂牌，市级行政审批流程再造迈出实质步伐，行政审批效率提高60%。实行“多证合一”改革，新增市场主体3.96 万户。市属31 家国有破产企业遗留问题基本解决，安置职工1.19 万人。国控集团及下属六大产业集团全部实行年薪制，资本运营机制进一步完善，直接支持发展资金55.5 亿元，撬动社会资本126.9 亿元。新增上市企业10 家。市级9 家公立医院实行药品零差率销售。大力实施科技创新，新增院士工作站6 家、高新技术企业37 家，实现了倍增目标；科技型中小企业新增427 家、达到1317 家。农村综合改革扎实推进，农村土地承包经营权确权基本完成，土地流转率达到32.1%。农民专业合作社实现村级全覆盖，双滦区农村集体资产股份制改革经验在全国推广。国有林场改革、供销社改革、城管综合执法改革走在全省前列。

民生保障水平得到新提升。坚持以人民为中心的发展思想，尽心竭力改善民生福祉。十四届人大一次会议上向全市人民承诺的“十项民生工程”20 件惠民利民实事全部完成。财政民生投入占公共财政预算支出的85%，达到281.1 亿元。城乡居民人均可支配收入达到27042元和9682 元，分别增长8.8%和10.8%。新增城镇就业5.48 万人，高校毕业生就业率达到95.4%， 城镇登记失业率3.53%。全市新建、改扩建各类学校52所，中心城区新建中小学校15 所，隆化县、兴隆县义务教育均衡发展通过国家验收，双桥区通过省级验收，义务教育均衡发展水平进一步提高。城乡居民医疗保险、养老保险参保率分别达到95%和99%，为14 万失地农民解决养老保险遗留问题。中心城区长期护理保险试点启动实施。改造棚户区6668 套、农村危房2.7 万户，95%的房地产开发遗留问题得到解决。启动实施基层医疗卫生服务“雨润工程”，市县34 家医院建立对口帮扶协作关系。承德民族团结清文化展览馆和博物馆、冰上运动中心等文化体育设施主体完工，“21 世纪避暑山庄”文化旅游产业园跻身全国首批示范园区创建行列。安全生产、食品药品安全形势保持平稳，法治承德、平安承德建设扎实推进，社会和谐稳定局面进一步巩固。

一年来，我们坚决落实全面从严治党各项部署，深入开展“两学一做”学习教育，认真执行中央八项规定和实施细则，驰而不息纠正“四风”，扎实推进微腐败专项整治和“一问责八清理”专项行动，加大行政监察和审计监督力度，进一步巩固强化反腐败斗争压倒性态势，政府系统作风建设取得显著成效。我们忠实履行宪法和法律赋予的职责，自觉接受人大法律监督与工作监督、政协民主监督以及社会监督、舆论监督，共办理人大代表建议281 件、政协提案357 件，按时办复率均为100%。我们进一步巩固军政军民团结局面，国防动员、优抚安置工作不断加强，荣获全省双拥模范城“九连冠”。人口计生、民族宗教、新闻出版广电、外事侨务、人民防空、史志档案、统计调查、气象地震、防灾减灾、对口援疆、邮政通信、妇女儿童、老龄、残疾人等工作取得新成绩。

各位代表，过去一年全市经济社会发展取得的成绩和进步，是习近平新时代中国特色社会主义思想领航定向、伟大实践的结果，是省委、省政府和中共承德市委正确领导的结果，是全市人民勠力同心、拼搏进取的结果。在此，我代表市人民政府，向全市383 万人民，向人大代表、政协委员，向各民主党派、工商联、无党派人士和人民团体，向驻承人民解放军、武警部队官兵和政法干警，向一直以来关心和支持承德经济社会发展的国内外各界朋友，致以崇高的敬意和衷心的感谢！

各位代表，在看到来之不易成绩的同时，我们也清醒地认识到，发展中还存在不少差距和问题，必须时刻保持强烈的责任感、紧迫感和忧患意识。主要是：经济总量小、底子薄、基础差的基本市情尚未根本改变，补短板、强弱项任务繁重；产业结构不合理，壮大实体经济尤为紧迫，经济发展质量亟待提升；贫困面大、贫困程度深，脱贫攻坚任务十分艰巨；科技创新水平不高，高层次人才匮乏，企业以创新引领发展的内生动力不强，新旧动能转换迫在眉睫；公共服务基础设施欠账较多、能力偏弱，社会治理、社区管理需进一步加强；安全生产、环境保护、金融秩序、政府债务、社会稳定等方面风险不容忽视，应对的压力和挑战较大；营商环境不优，开放水平不高，一些工作人员不担当不作为，形式主义、官僚主义等四风问题和消极腐败现象还不同程度存在，政府服务能力、质量和效率尚需大力改善。对这些问题，我们决不回避、决不推脱，一定拿出更加精准有力的举措认真加以解决，以新的发展成效回应全市人民的期盼和重托。

二、2018 年工作总体要求

今年是全面贯彻党的十九大精神的开局之年，是改革开放40 周年，是决胜全面建成小康社会、实施“十三五”规划承上启下的关键一年，做好今年工作至关重要。中国特色社会主义和经济发展进入新时代，承德的发展站在了新的历史起点上。我们一定要坚决贯彻习近平新时代中国特色社会主义思想，一定要坚决落实习近平总书记对承德作出的“定位于建设京津冀水源涵养功能区，同步考虑解决京津周边贫困问题”重要指示，一定要大力弘扬“牢记使命、艰苦创业、绿色发展”的塞罕坝精神，牢牢把握历史性窗口期和战略机遇期，乘势而上，只争朝夕，团结奋斗，用实干实政实绩书写贯彻落实党的十九大精神的生动篇章。

进入新时代，我们必须践行新理念、把握高质量发展新要求。新时代的发展，是追求更高质量、更有效率、更加公平、更可持续的内涵式发展，是承德摆脱传统路径依赖、实现跨越赶超的转型发展。我们必须牢固树立大质量观，让新发展理念深深植根于头脑、落实于行动，使创新成为第一动力、协调成为内生特点、绿色成为普遍形态、开放成为必由之路、共享成为根本目的，切实把高质量发展贯穿于经济社会发展各领域、全过程，以高质量发展引领转型升级，推动承德经济爬坡过坎、破茧成蝶。

进入新时代，我们必须肩负新使命、提升绿色发展新境界。承德作为京津冀水源涵养功能区和全国全省脱贫攻坚主战场，做优生态、全面脱贫两大政治任务历史地落在了我们肩上。我们必须牢固树立生态优先的发展理念，调高思想标尺和工作标尺，正确处理生态与发展之间的关系，坚持绿色发展要求，摒弃粗放落后的发展方式，用实际行动践行“两山”理论，坚定不移走生态优先、绿色发展之路，筑牢承德永续发展的根基命脉。

进入新时代，我们必须直面新矛盾、回应人民群众新期待。承德作为革命老区、民族地区和欠发达地区，发展不平衡不充分问题较为突出，城乡群众在就业、上学、看病、社保等方面还有不少操心事、烦心事，公共服务的能力和水平与人民群众对美好生活的期待相比还有不小的差距。我们必须始终把人民群众放在心中最高位置，把人民群众是否拥护、赞成和满意作为检验工作的最高标准，下大力解决好人民群众的现实困难，维护好人民群众的切身利益，一件事接着一件事抓、一年接着一年干，不断提高人民群众的获得感、幸福感。

进入新时代，我们必须抢抓新机遇、创造担当干事新业绩。党的十九大吹响建设社会主义现代化国家的前进号角，催人奋进；党中央实施京津冀协同发展、扶贫攻坚、生态文明建设等重大国家战略给我们带来的历史机遇，千载难逢；省委省政府支持承德走生态保护与绿色发展双赢之路，建设生态文明先行示范区，力度空前；塞罕坝建设者所创造的人间奇迹、所展现的时代精神就在我们身边，鼓舞斗志；承德政通人和万事兴的良好政治生态业已形成、区位和交通优势日益彰显。只要我们振奋精神，抢抓机遇，奋力拼搏，就一定能够谱写出无愧于时代、无愧于历史、无愧于人民的辉煌篇章！

今年政府工作的总体要求是：全面贯彻党的十九大精神，坚持以习近平新时代中国特色社会主义思想为统领，认真落实习近平总书记对河北、对承德作出的重要指示要求，按照省委九届六次全会和市委十四届三次全会总体部署，坚持稳中求进工作总基调，坚持高质量发展，坚持生态优先、绿色发展，紧紧围绕“12345”总体思路目标和“三区一城”发展定位，深化供给侧结构性改革，加快建设现代化经济体系，奋力开创新时代“生态强市、魅力承德”建设新局面，为打赢脱贫攻坚硬仗、决胜全面建成小康社会、谱写中华民族伟大复兴中国梦的承德篇章奠定更加坚实的基础。

今年全市经济社会发展的主要预期目标是：生产总值增长6.8%，规上工业增加值增长5%，服务业增加值增长10%以上；全部财政收入和公共财政预算收入均增长7%；固定资产投资增长6.5%；社会消费品零售总额增长11%；出口总值和实际利用外资均增长5%；城乡居民人均可支配收入分别增长8%和10%；PM2.5 平均浓度、地表水环境质量及化学需氧量、二氧化硫、氨氮、氮氧化物减排完成省下达目标，万元生产总值能耗下降4%；城镇登记失业率控制在4.5%以内。

上述目标，既符合高质量发展的时代要求，与我市“十三五”规划目标相衔接，又为调整优化经济结构留出空间，符合中央和省对承德的定位，符合我市的发展实际。我们要解放思想，奋发作为，撸起袖子加油干，苦干实干拼命干，努力创造新时代承德发展的崭新业绩！

三、2018 年重点工作

实现全年工作目标，必须统筹兼顾、把握关键，以重点突破活跃带动全局。着力抓好十个方面工作：

（一） 大力推动经济高质量发展。坚持质量第一、效益优先，以高质量发展引领转型升级，以高质量发展打造竞争新优势，以高质量发展赢得未来。

以高质量投资增强发展后劲。把项目建设作为重中之重，深入实施重点项目建设“三年攻坚行动”和“四个一”工程，谋划储备重点项目1000 个以上，重点推进超10 亿元项目100 个以上，实施超亿元项目1000 个以上，完成投资1200 亿元以上，绿色产业项目投资占比达到60%以上。落实落细“六个一”包保、“七人小组”“工作专班”和“通报约谈”等项目建设工作机制，着力保障重点项目土地供给，着力净化项目周边环境，着力提高土地“亩均效益”。推动特变电工、北京热力等一批重大项目落地，中电信息产业园、崔梨沟滑雪度假小镇加快开工建设，以高质量投资支撑全市经济实现量的增长、质的提升。

以高质量服务壮大实体经济。着力支持以绿色制造业为核心的实体经济加快发展，推进经济结构优化调整，制定出台支持实体经济发展的若干措施，推动资金、技术、人才等生产要素向实体经济集聚。落实国务院“降税、减证、提标”三项措施，降低实体经济交易成本。实施100 家重点骨干企业帮扶行动，大力优化企业发展环境，切实为企业发展解难题、办实事。力争新增规上工业企业40 家、“专精特新”中小企业12家、市场主体2.8 万户。围绕我市优势产业组建行业协会，增强企业契合发展优势。加强企业家队伍建设，组织企业家到发达国家和地区培训，开阔视野，提高素质。制定鼓励民间资本投资政策措施，力争民营投资占固定资产投资比重达到75%以上，民营经济增加值增长6.5%以上，让民营经济活力充分迸发。

以高质量供给扩大消费需求。深入开展增品种、提品质、创品牌专项行动，在中高端消费、绿色低碳、共享经济、现代供应链等领域培育新增长点，着力增强消费对经济发展的基础性作用。加快发展现代物流、金融保险等生产性服务业和医疗康养、便民商业等生活性服务业，推进怡达集团、远通集团等9 个京津冀一小时流通圈项目建设。加快实施电子商务进农村进社区和“互联网+流通”战略，扶持梵纳瑞等跨境电商做大做强，推动60%以上大型零售企业实现线上线下融合发展，鼓励“大槐树”等电商企业扩大农产品在线交易，全市农产品上行规模增长10%以上，电子商务交易额增长20%以上，服务业对经济增长的贡献率达到67%以上。

（二） 大力培育现代化经济体系。以培育壮大十大绿色产业为主抓手，积极探索构建现代化经济体系的机制和路径，力争绿色产业增加值占GDP 比重再提高5 个百分点、达到40%以上，绿色产业竞争力进一步增强。

推动传统产业迈向中高端。制定实施加快推进工业转型升级的指导意见，与深圳合作建立市级“工业设计”中心，打造“设计+”产业链。实施新一轮亿元以上技改升级项目80 个，传统工业向数字化、网络化、智能化、绿色化转变。强力推进钒钛产业向中间产品、钒钛新材料发展，支持承钢钒钛产业园、天大钒业高端合金、建龙钒氮合金等项目快速推进，加快产业链条向绿色化高端化延伸，提高国际国内市场占有率，钒钛产业主营业务收入达到450 亿元。强力推进装备制造业智能化发展，培育壮大军民融合产业，推动输送装备、智能仪器仪表等传统产业做优做强，支持比亚迪新能源汽车等龙头企业扩大产能、开拓市场，装备制造业产值增速达到10%以上。强力推进新型建材产业产学研一体化发展，提高集成服务、链式配套能力，力争产值达到200 亿元。强力推进绿色食品及生物医药产业发展，支持平安高科、天原药业等企业扩规模增效益，农产品加工业总产值增长8%以上。

推动新兴产业撑起新天地。围绕建设京津冀大数据综合试验区和国家绿色大数据中心，进一步完善大数据基础设施，建成与北京大数据直通直连网络。大数据产业从存储向研发、应用延伸，构建完整的大数据产业链条。加快德鸣大数据产业园、中经承园等重点项目建设，大数据产业产值实现倍增。围绕建设“中国高锶矿泉城”，做大做强本土企业，积极引进品牌企业，提高矿泉水产业产品销售量、市场占有率、品牌影响力，新增产能100 万吨。围绕建设国家级清洁能源产业基地，推动风电基地二期项目建成投产，加快丰宁抽水蓄能电站工程建设，清洁能源电力装机达到700万千瓦以上，发电量达到100 亿千瓦时。围绕建设京津冀节能环保产业基地，加快高新区节能环保产业园建设，节能环保产业产值达到100 亿元以上。

推动文化旅游产业再升级。瞄准国际旅游城市建设目标，加快建设“一核、两带、五大板块、六个旅游度假区”，双桥、滦平等6 个县区完成全域旅游示范区创建任务，为2019 年我市建成国家全域旅游示范城市奠定坚实基础。举全市之力承办好第三届河北省旅游产业发展大会，重磅推出“国家1 号风景大道”承德旅游金字招牌，高水平建设中国马镇、契丹部落等六大组团20 个精品项目，倾力打造“木兰秋狝大典”“马秀”两台大型实景演出，实现精彩、震撼、超越的目标。下大力完善旅游基础设施，培育航空体验、房车营地、VRAR 等新兴旅游业态。金山岭长城建成国家5A 级景区，兴隆山等一批景区提档晋级。新建游客服务中心27 个、旅游厕所200个、停车位2 万个以上。深度实施“旅游+”战略，加快发展文化创意、影视演艺、数字传媒等文化产业，国家图书馆文献战略储备库开工建设，承德北京影视基地等一批重大项目落地实施。打造宽城满族风情、兴隆塞外江南、营子山楂小镇等23 个品牌特色小镇。组建承德演艺集团。举办中国承德非物质文化遗产大展。大力发展冰雪运动，积极承办重大冰雪赛事，承德市冰上运动中心建成投用，文化旅游业对经济增长的贡献率进一步提升。

（三） 大力推进精准扶贫精准脱贫。以更大的决心、更实的举措、更严的要求，进一步深化“八措并举”组合拳，推动脱贫攻坚向纵深发展，确保11 万贫困人口实现稳定脱贫，兴隆、滦平、承德县摘帽出列。

向重点区域人群聚焦发力。持续加大对丰宁、围场、隆化3 个深度贫困县72 个深度贫困村的支持力度，扎实开展基础设施和公共服务提升工程。积极推进易地扶贫搬迁“两区同建”，确保去年开工的52 处集中安置小区今年全部竣工。加大政策兜底保障力度，在巩固提升“632”医疗保障救助扶贫成果基础上，探索引入商业保险，进一步减轻群众医疗负担，让贫困群众就医有兜底、看病不再愁。

向特色扶贫产业聚焦发力。加快打造“五个百万基地”“一环六带”扶贫产业带，着力发展林果、蔬菜、食用菌、中药材等九大特色扶贫产业，继续推进以肉牛、马铃薯等产业为主导的跨县连片扶贫产业聚集区建设，加快推广“互联网+扶贫”新模式，培育扶贫龙头企业610 个，扶持产业扶贫示范村80 个、乡村旅游贫困村120 个，新增村级光伏扶贫电站645 个、户用光伏1.5 万个，确保贫困村、贫困户产业项目全覆盖。

向创新扶贫机制聚焦发力。完善社会帮扶机制，充分发挥中省直、京津及唐山市对口帮扶作用，选优派强190 个市直和563 个县市区直驻村工作队。巩固金融扶贫机制，“政银企户保”实现全覆盖。建立长效机制，扶贫与扶志、扶智相结合，激发贫困群众脱贫致富的内生动力。强化责任机制，深入开展扶贫领域腐败和作风问题专项治理，严格资金和项目全程监管，严格评估考核和追责问责，使扶贫工作得到群众认可、经得起历史检验。

（四）大力实施乡村振兴战略。紧紧围绕“产业兴旺、生态宜居、乡风文明、治理有效、生活富裕”总要求，加快编制乡村振兴总体规划和实施方案，实现乡村振兴战略开好局、起好步。

着力提升现代农业。大力推进农业产业化，加快构建“龙头企业+基地+园区+合作社+农户”的农业产业经营机制，培育市级以上龙头企业50 家，实施千万元以上产业化项目160 个，大幅提高农业综合效益。突出发展科技农业，积极争创国家农业高新技术产业示范区，转化落地农业新技术成果20 项以上，构建全产业链技术支撑体系。突出发展绿色农业，加快创建省级农业绿色发展示范市，扩大绿色有机产品规模，新增高效节水灌溉面积9 万亩。突出发展质量农业，健全质量追溯体系和监管体系，农业标准化生产覆盖率达到60%，平泉、双滦建成国家级农产品质量安全县（区）。突出发展功能农业，立足建设全国功能农业扶贫综合示范区，打造功能农业田园综合体，加快建成辐射京津冀、一二三产融合发展的功能农业“智慧中心”。力争新增国家地理标志保护产品11个。加快种植结构调整步伐，年内调减籽粒玉米30 万亩。

着力打造美丽乡村。深入实施农村人居环境整治三年行动计划，加强农村建设规划管控，保护乡村风貌，开展田园建筑示范，新建一批高水平的美丽乡村精品片区。多渠道增加投入，补齐农村基础设施和公共服务短板，新改建农村公路900 公里，实施农村电网改造项目785 个，推进行政村4G 网络全覆盖，农村垃圾处理率达到90%，卫生厕所普及率达到57%以上，行政村主街道硬化率达到100%。建管并举，建设广大农民安居乐业的美丽家园。

着力培育新型农民。加强农村思想道德建设，促进形成良好家风、淳朴民风、文明乡风。发挥妇联、共青团等群团组织和农村红白理事会等各类基层群众自治组织作用，通过修订完善村规民约，开展丰富多彩、形式多样、喜闻乐见的群众性活动，培树一批文明家庭和文明个人，教育引导群众自觉抵制不孝敬不赡养老人、婚丧嫁娶大操大办、索要高额彩礼、封建迷信等不良风气，传承弘扬中华民族传统美德。加强农民职业技能培训，培养新型职业农民，提高农民增收致富本领。支持大中专毕业生和农民工返乡创业，为新农村建设培养一批骨干和带头人。

（五） 大力推进生态文明建设。承德作为塞罕坝精神的发源地，生态文明建设必须自我加压、调高标尺，持续巩固放大生态优势，努力建设国家生态文明先行示范区。

坚定不移打好水环境整治攻坚战。以全面落实市县乡村四级“河长制”为总抓手，坚决落实国家“水十条”、省“水五十条”，标本兼治、长短结合，全面改善和提升水环境质量。采取污水处理厂增能提质、垃圾整治、治企断源、舍饲禁牧、园区养殖、粪便集中处理、绿色种植、河道修复等综合措施，实施水污染防治“三年百项重点工程”，今年开工治理项目48 个。完善上下游联防联控机制，实行“一断面一策、一源一档”管理，推行第三方治理，完成全市13 个国家考核地表水环境质量自动监测站建设，确保水环境质量“长治久清”。

毫不放松打好蓝天保卫战。制定实施大气污染防治三年作战计划和冬季清洁取暖三年规划，严格落实“1+18”政策体系，开展太阳能、地热、电代煤气代煤等清洁方式试点，新增集中和清洁供热面积300 万平方米，环境空气质量自动监测站实现乡镇全覆盖，PM2.5 平均浓度控制在35 微克以下。

一招不让打好国土治理持久战。开展国土绿化三年行动，深入实施京津风沙源治理、京冀津冀水源林、山水林田湖修复试点等重大生态项目建设。继续推进国家绿色矿业发展示范区建设“6743”工程，造林绿化65 万亩，治理水土流失面积500 平方公里。实施生态保护红线勘界定标，加强风景名胜区和自然保护区管理。严格落实“土十条”，建立固体危险废物全过程监管制度，启动化肥农药零增长行动，加强农业面源污染防治，全力维护土壤环境安全。

积极创新完善生态文明体制机制。发挥塞罕坝全国生态文明建设范例的示范作用，编制实施《塞罕坝机械林场及周边地区可持续发展规划》，进一步提升坝上地区生态建设水平和综合发展能力。围绕《水源涵养功能区保护条例》 的颁布实施，深化自然资源资产负债表应用，开展绿色GDP 统计试点，进一步扩大领导干部自然资源资产离任审计试点范围。推动与京津建立多元化生态补偿机制，探索用能权、碳排放权、排污权等生态产品交易模式，打造辐射京津冀的生态产品交易市场。大力推进绿色循环低碳发展，促进形成绿色生产方式和生活方式。加强环境保护执法监管，严厉打击环境违法违规行为，坚决守住生态保护底线，为承德永续发展创造和积累更加雄厚的生态财富。

（六）大力拓展协同发展广度深度。坚持立足京津、面向全国、放眼世界，统筹区域布局，优化要素配置，以全方位开放促进高质量发展。

深层次推进京津冀协同发展。以落实津承“1+4”和京承战略合作协议为重点，瞄准文化、旅游、体育、会议展览、教育医疗等优势资源疏解，深化与京津的全方位对接合作，谋划和承接产业合作项目80 个以上，新落地50 个以上。加快津冀六沟产业园、丰宁怀丰产业园建设，推动承德朝阳产业园、双滦北京大洋路批发市场等项目落地实施。天津中德应用技术大学承德分校正式招生。

全方位扩大对内对外开放。坚持引进来走出去，紧盯长三角、珠三角和港澳台，面向“一带一路”国际市场，跨出小天地、构建大格局。深入开展“2018 开放招商年”活动，制定出台扩大对外开放的实施意见，完善产业链招商指导目录，推动组织天津投资贸易洽谈会、百家名企进承德等专题招商活动，组建深圳承德企业商会，引进市外资金380 亿元以上。积极拓展对外贸易，引导和推动企业“走出去”，支持平泉保税物流中心、双滦鹏涛智慧云谷建设，打造2个县域特色产业外贸基地，新增出口备案企业30 家。

大力度提升开发区承载能力。深入实施开发区改革发展三年攻坚行动，深化体制机制改革，全面提升基础配套能力，探索建立园区共建共享机制。支持各县市区围绕十大绿色产业，选准产业门类，构建特色突出、区域联动、错位发展的园区格局，开发区新入园企业170家以上，固定资产投资增长20%以上，经济总量占到全市的“半壁江山”。

高标准提升新型城镇化质量。大力推进以人为中心的新型城镇化，促进产业、人口向城镇集中，全市城镇化率提高1.2 个百分点以上。启动新一轮城市总体规划修编。增强中心城区辐射能力，深入实施“一带三区”城市发展战略，谋划推进高铁商圈一期等重点产业和基础设施项目30 个。双峰寺水库启动蓄水，临空经济区2 平方公里起步区开工建设。加快县城和小城镇建设，持续开展县城建设攻坚行动，实施“一补三化”15 项重点任务，建设一批城市排水防涝设施、地下综合管廊和海绵城市示范工程。推进城乡交通基础设施建设，完善承德机场功能，增开2～3 条新航线、通达10 个以上城市，吞吐量突破30 万人次。京沈高铁承德段具备通车条件，承平高速全面开工建设。全力推进津承城际铁路、承克高速前期工作，开工建设干线公路530 公里，加快构建现代综合交通体系。

高品质推进文明城市创建。深入实施以国际旅游城市建设为统领的“五城联创”，启动全国文明城市创建三年行动计划，围绕“八大环境”建设补短板、强弱项、提品质、增内涵，确保2020 年全国文明城市创建成功。加快补齐公共服务设施短板，今年启动中心城区游客服务中心、市图书馆建设，承德民族团结清文化展览馆和博物馆建成投用。加快智慧城市建设，提升城市精细化、智能化管理水平。完成城市市容和环境卫生管理地方立法相关工作，严厉整治占道经营、城市“牛皮癣”等乱象。深入开展道路交通秩序整顿，实施道路改扩建、优化交通组织、强化交规教育、健全志愿者队伍、加强停车场管理、错时上下班等措施，多措并举、综合施策解决交通拥堵问题。着力提升社区建设管理水平，改善社区办公条件，提高社区工作人员待遇。推行楼长制、路长制，织密城市基层管理网络，服务群众，方便百姓，架起党委政府和群众之间的“连心桥”。培育和践行社会主义核心价值观，广泛开展文明礼仪规范、公共场所治乱等“十个专项行动”。深入挖掘历史文脉、民族特色和地域风情，厚植承德文化传承，支持市评剧团、歌舞团改革发展，引进高雅艺术文化产品，培育文化消费市场，让文明之花在承德大地更加璀璨。

（七） 大力实施创新驱动提升发展内生动力。坚持把创新作为引领发展的第一动力，推动各类创新资源加速向承德聚集。

积极培育创新主体。把人才培养引进作为高质量发展的当务之急，制定出台含金量高、吸引力大的优惠政策，实施引进高端人才“千人计划”，今年引进300 名以上。全面落实“1+9+N”系列科技创新政策，引导企业加大研发投入力度。实施科技型企业培育“331”工程，新增高新技术企业30 家、科技型中小企业300 家、科技小巨人企业10 家。万人发明专利拥有量达到1.29 件，高新技术产业增加值增长12%以上。

积极打造创新平台。围绕协同创新，与京津共建院士工作站、工程技术研究中心、产业技术研究院和众创空间等科技创新平台15 家以上。聚焦战略性新兴产业，实施科技成果转化项目10 个，新增省级产业技术创新战略联盟1 家。支持平泉市、承德县省级创新型县城试点建设，积极争创国家可持续发展议程创新示范区。

积极推进质量强市。制定加快质量强市建设实施意见，推动标准、质量、品牌、信誉联动提升，以高标准、高质量、名品牌构建承德信誉。组建质量与品牌协会、标准化专业技术委员会，培育一批有影响力的标准领跑企业。开展旅游业、制造业、食品农产品等质量提升“十大专项行动”，夯实质量基础工作。积极争创省政府质量奖，评选颁发市政府质量奖。争创中国驰名商标3 件、中国地理标志商标5 件、省级以上知名品牌2 项、省级名优产品30 项，双滦检验检测园争创国家级公共检验检测平台。

（八）大力推进重点领域和关键环节改革。坚持上下联动、精准发力，坚定不移进行重点领域和关键环节改革，着力破解影响发展的体制机制障碍。

以最大决心和力度优化营商环境。大力开展“承德办事一次成”改革，作为各级政府“一把手”工程强力推进实施，不达目标不收兵。完善提升“互联网+政务服务”平台功能，编制“承德办事一次成”清单，全面推开“在线咨询、网上审批、快递送达”服务模式，年底前一次办结事项占许可事项的60%以上。深化“放管服”改革，继续向基层下放一批行政权力，“三级清单、两个代办”实现全覆盖。深化相对集中行政许可权改革，加快行政审批标准化规范化建设，市县两级行政审批局全部规范运行。深化“双随机、一公开”监管，探索建立营商环境评价体系，重拳铁腕治理不作为、乱作为、慢作为，让市场主体感到营商环境显著改善，让投资者来得放心、干得顺心、发展得有信心。

以提高效益为核心深化国有资产管理改革。改革国有资本授权经营体制，健全和完善国控集团及产业平台企业领导人员管理体制和绩效考核、薪酬分配制度。进一步完善国有资产监管体制机制，向管理资本为主转变，切实提高国资监管的针对性、有效性和系统性，促进国有资产保值增值。全面推进行政事业单位国有资产管理改革，整合财汇、财通、蓝天等基金，组建承德投资集团，进一步健全国有资本运营体系。

以优化管理为重点促进财税金融改革。围绕保民生、促发展优化财政支出结构，深化绩效预算管理制度改革，推进财政事权和支出责任划分，建立财源建设新机制，稳妥推进水资源税征收和环保税改革。支持承德银行在雄安新区和张家口设立分支机构，兴业银行承德分行开业运营，组建兴隆、隆化村镇银行，鼓励支持金融机构向县市区和乡镇社区布点。支持企业借助资本市场扩大融资，启动承德热力公司主板上市前期工作，新增上市企业6 家，金融业增加值达到150 亿元以上。

以激活资源资产为突破深化农村综合改革。全面完成农村承包地确权登记颁证工作。推进承包地“三权分置”改革，促进适度规模经营，土地流转达到150 万亩、流转率达到36%。积极稳妥推进农村集体产权制度改革，所有行政村全面完成农村集体资产清产核资工作。盘活农村各类资源资产，推进农村产权流转交易，探索“市民农宅”等新型模式。加快培育农业新型规模经营主体，新认定省级农村股份合作制经济组织10家以上、市级100 家以上。加快农场、林场、牧场办社会职能改革，全面完成32 个国有林场改革任务。

统筹推进社会事业改革。深化教育领域综合改革，围绕落实党中央、国务院关于深化教育体制机制改革和全面深化新时代教师队伍建设改革的意见，统筹推进学前教育、城乡义务教育、高中阶段教育、职业教育、高等教育和特殊教育发展，实施名校、名师、名校长工程，提高人民群众对教育的满意度。深化医药卫生体制改革，持续推进“雨润工程”，探索多种形式医联体建设，实行公立医院人员编制备案管理改革，更好保障城乡群众病有所医。扎实推进住房制度改革，坚持“房子是用来住的、不是用来炒的”定位，探索建立多主体供给、多渠道保障、租购并举的住房制度，促进房地产市场平稳健康发展。

（九） 大力防范和化解重大风险。坚持守土有责、守土尽责、守土负责，牢牢掌握风险防控主动权，进一步筑牢首都政治“护城河”。

有效防控金融风险。深入开展全面彻底的金融领域风险隐患排查处置专项行动，大力整顿规范金融秩序，严厉查处非法集资、金融诈骗等违法行为，有效防范化解信用风险和地方金融机构资管业务风险。建立金融风险监测、预警和处置机制，规范小额贷款公司、担保公司和农民专业合作社等经营行为，坚决守住不发生系统性、区域性金融风险的底线。

高度关注政府债务风险。严格执行地方政府债务限额管理制度，坚决禁止通过政府担保、PPP、购买服务等方式变相违规举债，完善政府债务管理台账，完善风险防控措施，坚决管住不产生新的隐性债务。按照量力而行的原则实施基础设施建设，坚决防止盲目随意铺摊子上项目，坚决防止变相扩大政府债务搞形象工程、政绩工程，确保财政安全。

严管严控安全风险。全面推进安全生产领域改革发展，严格落实企业主体责任、地方属地管理责任、部门监管责任。继续推进重点行业领域安全风险分级管控和隐患排查治理，大力加强重点行业领域专项治理。进一步加大执法监察力度，严防各类事故发生。强化食品药品全过程监管，实施国家食品安全示范城市创建“六大建设工程”，确保人民群众舌尖上的安全。

重拳出击扫黑除恶。坚决有力开展扫黑除恶专项斗争，严查狠打黑恶势力犯罪活动。严厉打击涉枪涉爆、电信诈骗、涉众型经济犯罪等违法犯罪行为，完善立体化、信息化社会治安防控体系。构建新型矛盾排查化解机制，妥善处理各类突发事件，切实保障人民群众生命财产安全，维护社会安全稳定，打造法治的承德、平安的承德、和谐有序的承德。

加强全民国防教育、国防动员和后备力量建设，做好退役人员安置和管理服务工作，巩固军政军民团结的好局面。充分发挥工会、共青团、妇联等人民团体和群众组织桥梁纽带作用。继续做好人口计生、民族宗教、新闻出版广电、外事侨务、人民防空、史志档案、气象地震、地理信息、对口援疆、邮政通信、妇女儿童、老龄、残疾人等工作。

（十） 大力办好事关人民群众切身利益的民心实事。民有所呼、我有所应，我有所为、民有所享。通过向社会公开征集，充分考虑群众所需所盼所想，本着量力而行、尽力而为的原则，今年集中力量实施“十大民心工程”，再办20 件民心实事。一是教育优先发展工程。①实施学前教育三年计划，全市新建改扩建公办幼儿园52 所，新增学位4500 个，建成普惠性幼儿园20 所，其中中心城区7 所，进一步缓解入园难、普惠园少问题。②全市新建改扩建义务教育中小学校60 所，其中中心城区13 所，市特教学校启动迁建，世纪城学校、双峰寺镇中学、西地满族中学、新民族小学和双滦区第六小学5 所学校投入使用，增加学位1.12 万个， 有效缓解“大班额”问题。③中心城区中小学校全面推行课后延时服务，逐步解决学生家庭负担重问题。二是社会保障提升工程。④城乡居民年低保标准提高到7200 元和3900 元，分别高于省定标准600 元和300 元。⑤中心城区符合参保条件的24 万城镇职工实现长期护理保险全覆盖，承德县和营子区试点参保5 万人以上。三是医疗健康雨润工程。⑥多措并举提高基层医疗卫生服务能力，组织市县医疗机构400 名医师开展下乡服务，实施千名基层卫生人才引进培养计划，对服务千人以下行政村的村医每年给予5000-15000 元定额补助。四是困难群众安居工程。⑦ 全市新改造棚户区8010 套，其中中心城区完成2735 套，大榛子沟后山、鹿栅子沟等区域纳入改造范围。⑧实施农村危房改造1.1万户。⑨推进老旧小区改造提升，对中心城区双柳、万树园、中兴路、麻纺厂等58 个小区实施“管网改造、路面硬化、楼顶防水、外墙保温、道路照明”综合改造。⑩中心城区实现物业管理全覆盖，物业标准化管理达到30%以上，3 年内达到100%，让人民群众在住有所居中创造新生活。五是环境净化工程。⑪全市完成“双违”拆除100 万平方米，双桥区50 万平方米违规建筑、违法占地全部“清零”。⑫全部取缔中心城区主次干道占道经营。六是中心城区畅通工程。⑬启动101 国道改线，新建改建广仁大街、水泉沟路等城市道路11 条，打通碧峰门、旱河南路一期、开发东区中路、嘉和广场南路、偏岭子大桥东路5 条“断头路”。⑭新增纯电动公交车100 台，调整优化33 路、39路等6 条公交线路，在避暑山庄和外八庙景区周边新投放共享单车1700辆。⑮规范停车场收费服务管理，新增停车位2500 个，让群众出行越来越便捷。七是环保设施改善工程。⑯城市生活垃圾无害化处理率达到98、县城达到95以上，完成承德环能热电二期技术改造。⑰扩建改造太平庄、双滦区等7 个污水处理厂站，上板城等4 个污水处理厂站建成投用，新建改建污水管网43 公里。全部取缔武烈河市区段入河污水直排口。八是中心城区清洁供热工程。⑱下决心用三年时间彻底取缔中心城区散煤取暖，年内完成8000 户清洁供热改造，让百姓的屋更暖，让城市的天更蓝。九是文化惠民工程。⑲举办大型文艺演出季活动，邀请省级以上文艺团体来承演出3 场以上，组织市县文艺团体深入社区农村公益演出1000 场以上，让广大群众共享文化盛宴。十是平安承德工程。⑳新建改建派出所20个、社区（农村） 警务室60 个，新增视频监控摄像头3500 个，让老百姓踏踏实实、平平安安过日子。

各位代表，人民群众的小事就是我们的大事。我们一定把人民放在心中至高无上的位置，以造福人民为最大政绩， 坚决办好民心实事，坚决兑现民生承诺，让全市人民生活的更加安康、更加殷实、更加幸福。

四、努力建设人民群众满意的政府

打铁必须自身硬。新时代要有新气象，更要有新作为。全市政府系统必须坚持和加强党的全面领导，必须忠诚履职、诚实守信、勤勉尽责，必须持之以恒转作风、提效能、强本领，加快建设服务型政府、法治政府、效能政府、诚信政府和廉洁政府。

（一）牢固树立“四个意识”，坚定正确政治方向。坚决把维护以习近平同志为核心的党中央权威和集中统一领导作为最高政治原则和根本政治规矩，切实增强维护和捍卫核心的思想自觉、政治自觉和行动自觉，坚决贯彻中央、省委省政府和市委各项决策部署，始终在政治立场、政治方向、政治原则、政治道路上同党中央保持高度一致。推进“两学一做”学习教育常态化制度化，扎实开展“不忘初心、牢记使命”主题教育，坚定不移以习近平新时代中国特色社会主义思想武装头脑、指导实践、推动工作。礼拜五秘书网

（二）全面推进依法行政，切实维护公平正义。深入落实法治政府建设实施纲要，坚持把法治思维和法治方式贯穿于政府工作全过程，切实提升政府工作法治化水平。认真落实政府常务会议学法制度，推动法律顾问制度市县区全覆盖，积极推进行政负责人出庭应诉。严格规范公正文明执法，加快推进综合执法改革，依法维护人民群众和市场主体切身利益。自觉接受人大法律监督、工作监督和政协民主监督，广泛听取民主党派、工商联、无党派人士、人民团体意见，加大政务公开和信息公开力度，进一步加强公众参与平台建设，畅通民意反映渠道，让人民监督权力，让权力在阳光下运行。

（三）有诺必践遵约守信，倾力打造诚信政府。深入开展政务诚信建设年活动，建立政务和行政承诺考核机制。各级政府必须严格履行各项约定义务，承诺的事项和出台的政策必须坚决兑现，着力打造“诚信承德”形象品牌。建立司法公信责任制度，深入推行“阳光办案”“阳光执法”，畅通群众投诉和民意表达渠道，促进政府公开、公平、公正行使社会管理职能。健全失信惩戒机制，让失信的部门和人员受到惩戒，用政府的诚信引领社会信用体系建设，打造遵约践诺、诚实守信的承德新名片。

（四）转变作风实干实政，掷地有声狠抓落实。坚持深入实际调查研究，敢于直面矛盾，善于打攻坚战、啃硬骨头，练就有担当的宽肩膀，提高干成事的真本领。健全正向激励和容错纠错机制，坚决惩治“推绕拖”等懒政怠政行为，以永不懈怠的精神状态和一往无前的奋斗姿态推动各项工作落实，切实担当起党和人民赋予的新时代使命任务，做出经得起实践、人民和历史检验的实绩，交出成绩优异、人民满意的新时代答卷。

（五）从严治政廉洁从政，持之以恒正风肃纪。严格落实中央八项规定和实施细则精神，深入开展纠正“四风”十查十改活动，持之以恒克服形式主义、官僚主义，久久为功祛除享乐主义和奢靡之风。深入开展基层微腐败专项治理，把惩治“蝇贪”和扶贫领域腐败与扫黑除恶结合起来，严肃查处侵害群众利益的不正之风和腐败问题，做到有腐必反、有贪必肃。把践行“三严三实”贯穿于全部工作生活中，不断向廉洁自律的高标准看齐，做到心有所戒、行有所止，守住底线、不碰高压线，努力建设一支信念坚定、为民服务、勤政务实、敢于担当、清正廉洁的公务员队伍。

各位代表，美好生活是干出来的，幸福是奋斗出来的。让我们更加紧密地团结在以习近平同志为核心的党中央周围，高举习近平新时代中国特色社会主义思想伟大旗帜，认真贯彻落实中央、 省委省政府和市委的部署要求，不忘初心，牢记使命，忠诚担当，接续奋斗，为开启新时代生态强市、魅力承德建设新征程而努力奋斗！
